# Aldehyde dehydrogenase 1 isoenzyme expression as a marker of cancer stem cells correlates to histopathological features in head and neck cancer: A meta-analysis

**DOI:** 10.1371/journal.pone.0187615

**Published:** 2017-11-07

**Authors:** Yue Dong, Sebastian Ochsenreither, Chengxuan Cai, Andreas M. Kaufmann, Andreas E. Albers, Xu Qian

**Affiliations:** 1 Key Laboratory of Laboratory Medicine, Ministry of Education, Zhejiang Provincial Key Laboratory of Medical Genetics, Wenzhou Medical University, Wenzhou, P.R. China; 2 Hematology, Oncology and Tumorimmunology, Charité – Universitätsmedizin Berlin, corporate member of Freie Universität Berlin, Humboldt-Universität zu Berlin, and Berlin Institute of Health, Campus Benjamin Franklin, Berlin, Germany; 3 Clinic for Gynecology, Charité – Universitätsmedizin Berlin, corporate member of Freie Universität Berlin, Humboldt-Universität zu Berlin, and Berlin Institute of Health, Campus Benjamin Franklin, Berlin, Germany; 4 Department of Otorhinolaryngology, Head and Neck Surgery, Charité – Universitätsmedizin Berlin, corporate member of Freie Universität Berlin, Humboldt-Universität zu Berlin, and Berlin Institute of Health, Campus Benjamin Franklin, Berlin, Germany; Augusta University, UNITED STATES

## Abstract

There is a lack of predictive biomarkers that can identify patients with head and neck squamous cell carcinoma (HNSCC) who will experience treatment failure and develop drug resistance, recurrence, and metastases. Cancer stem-like cells (CSC) were identified as a subset of cells within the tumor in a variety of solid tumors including HNSCC. CSC are considered the tumor-initiating population responsible for recurrence or metastasis and are associated with therapy resistance. This meta-analysis including fourteen studies with altogether 1258 patients updates and summarizes all relevant data on the impact of ALDH1^+^ CSC on the prognosis of HNSCC and its association with clinicopathological parameters. ALDH1 expression is highly correlated with tumor differentiation (G3 vs. G1+G2; odds ratio = 2.85. 95% CI: 1.72–4.73, P<0.0001) and decreased overall survival (relative risk = 1.77. 95% CI: 1.41–2.22, P<0.0001) if one out of seven studies was excluded because of heterogeneity. These findings provide insights into the understanding of more aggressive tumor phenotypes and also suggest that the prognostic value provided by HNSCC-subtyping by CSC frequency warrant further clinical investigation.

## Introduction

Recent discoveries in cancer research continue to support the theory that cancer stem cells (CSC) initiate metastasis formation and tumor recurrence after initial therapy [1, 2]. Such a cancer initiating cell population has not only been described for head and neck squamous cell carcinoma (HNSCC) but also proved being relevant for clinical outcome of patients with such a disease as observed by our group and others [3–5]. HNSCC is regarded one of the most prevalent malignant diseases worldwide. Multimodal treatment strategies have substantially improved survival in patients with curative potential [6–9]. However, treatment decision in clinical routine especially for locally advanced (LA) HNSCC remains a challenge and is based on conventional factors such as tumor stage, comorbidities and a patient’s responses to ongoing treatments [10]. Among patients who had LA diseases and even with similar histopathological characteristics, diversity in responses to the same treatment regimen resulting also in differences in prognosis suggests so far unknown determining parameters [11]. Thus, additional stratification factors could augment the TNM staging system to improve prediction of long-term survival. That is, biomarker stratified treatment strategies could become a reality. Although HNSCC prognosis, and specifically in oropharyngeal cancer (OPSCC), has clearly been influenced by detection of human papilloma virus (HPV) infection [12], additional biomarkers are needed for HPV-negative tumors. Furthermore, in case CSC have a predictive or prognostic role for patients with HNSCC as suggested by preclinical studies, the targeting of the CSC cell population in combination with conventional therapies aiming at a reduction or eradication of the bulk tumor would be a reasonable approach to improve therapeutic efficacy by prevention of local recurrence or metastasis formation.

CSC can be defined by their biological features like unlimited proliferative capacity, and enhanced resistance to conventional treatment modalities like chemotherapy or radiation, by phenotypical markers like receptor expression, or by indirect detection of specific transmembrane transporter molecules. Among these CSC markers, the aldehyde dehydrogenase 1 (ALDH1) isoenzymes, which can be detected by their enzymatic function as well as by immunohistochemistry, are well studied in HNSCC. ALDH1 is associated with a stem cell-like phenotype, which is among other mechanisms a direct consequence of its obvious activity for oxidation of retinal to retinoic acid leading to cell differentiation [13]. Furthermore, ALDH1 expression enhances resistance to several anticancer drugs by its catalytic activity [14]. In cell lines, ALDH1-positivity is associated with tumorigenesis and resistance to chemo-radiation [4, 15]. ALDH1A1 positive CSCs are found both in primary tumors originating from the oral cavity, oropharynx, hypopharynx and larynx [5] and in lymph node metastases [16, 17]. Clinical studies have shown the prognostic value of ALDH1A1 in patients with LA HNSCC as well as in patients with nodal and visceral metastatic disease [5, 17].

Because of the persistence of CSC during therapy, which contributes to tumor relapse and its capacity for recapitulation of the heterogeneous features of a cancer, eradication of CSC is viewed as an important challenge for a successful cancer therapy. In a recent *ex vivo* study, NCT-501, a theophylline-based inhibitor of ALDH1A1, was able to overcome secondary resistance to several chemotherapeutic agents. The combination with cisplatin treatment was found to significantly decrease the proliferation of primary HNSCC cells compared to treatment with the individual agents [18]. Moreover, ALDH1A1 has been proven to be immunogenic and a target for cytotoxic T-cells. It has been shown that T-cell lines specific for MHC class I-restricted epitopes of ALDH1A1 can eliminate ALDH1A1 positive cancer cells in HNSCC [19, 20]. These observations imply a clinical usefulness of ALDH1A1 not only as a predictive biomarker but as potential target in HNSCC.

In order to assess clinical and biological characteristics such as histological appearance, lymph node metastasis and patient’s survival in relation to ALDH1 expression in HNSCC, we conducted a systematic review of the literature and meta-analysis. Strategies targeting ALDH1 positive CSC are also discussed.

## Materials and methods

### Literature search strategy and selection criteria

Electronic literature databases PubMed, EMBASE, the Cochrane Library, and WangFang databases for studies published before December 31^st^, 2016 were searched using the following key words: “ALDH1” or “aldehyde dehydrogenase 1” and “head and neck squamous cell” or “oral” or “laryngeal” or “pharyngeal” or “tongue” or “oropharyngeal” and “cancer” or “carcinoma” or “neoplasms”. Titles and abstracts were scanned to identify the candidate publications. Systematic reviews, letters, and case studies were excluded. Manuscripts from the reference lists of original or review articles were also screened for additional publications. In a second step, full-text articles were assessed to check for eligibility. To be included in the current study, original articles were required to meet the following criteria: (1) patients with HNSCC; (2) correlations between ALDH1 overexpression and histopathological parameters, overall survival (OS) or disease-free survival (DFS) of HNSCC patients were analyzed.

### Data extraction

Pivotal data were extracted from all eligible publications independently by two of the authors (DY and QX). For this, data tables were composed to extract all relevant data from texts, tables and figures including patient characteristics, tumor stages and additional clinical features together with clinical endpoints like OS and DFS. Additionally, for each article, the authors, the year of publication, and the number of patients were extracted. To digitalize and extract survival data from Kaplan–Meier plot curves, the software GraphClick (Version 3.0.2, Arizona Software 2010, http://www.arizona-software.ch/graphclick) was used.

### Study quality

The Newcastle-Ottawa Scale (NOS), a risk of bias assessment tool for observational studies, was used to evaluate the methodological quality of included studies [21, 22]. The quality assessment values ranged from 0 to 9 points. There are three categories including selection (4 points), comparability (2 points), and exposure (3 points) (S2 Table). The result was an overall risk of bias rating of each study and score ≥5 is considered high quality [22]. Two independent reviewers evaluated the risk of bias for each study.

### Statistical analysis

All statistical analyses were performed using the methods described in the Cochrane Handbook for Systematic Review of Interventions [23], using the Reviewer Manager Software, version 5.3 (Cochrane Library, Oxford, UK) and STATA 12.1 (StataCorp LP, Texas, USA). The impact of ALDH1 overexpression on dichotomous data was expressed as the odds ratio (OR) and risk ratio (RR) with 95% Confidence Intervals (CI). OR was used to assess the association between ALDH1 overexpression and clinicopathological parameters such as clinical stage including lymph node status and tumor histology. RR was used to assess the association between ALDH1 overexpression and outcome (OS and DFS) [24].

We assessed the statistical heterogeneity by observing I-squared (*I*^2^) and Q test [25, 26]. If a significant heterogeneity existed (*I*^*2*^ >50% or P<0.05), a random effect model was used to calculate OR and RR. Otherwise a fixed effect model was used [26]. In order to explore the possible sources of heterogeneity, subgroup analyses and sensitivity analyses were conducted. In addition, Egger’s test was used to evaluate the publication bias [27].

## Results

### Description of the included studies

**Fig 1:**
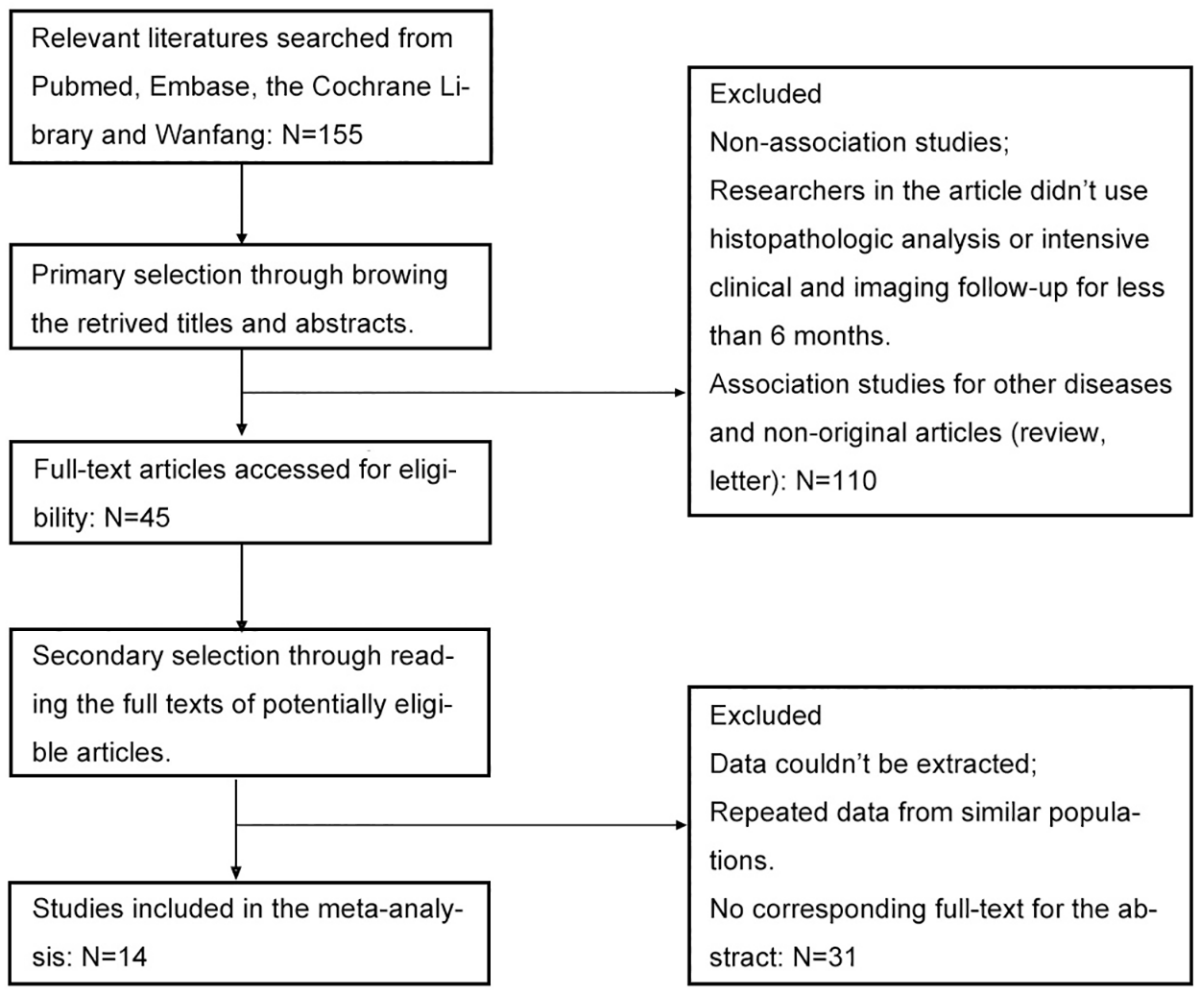
Flow-chart for identification of eligible studies.

155 studies were retrieved from the databases and 14 studies [5, 16, 17, 28–38] met all predefined inclusion criteria and therefore were included in the meta-analysis (Fig 1). The representative search strategies are illustrated in S1 File. The total number of patients included was 1258. The main characteristics of the eligible studies are summarized in Table 1. Ten articles provided clinicopathological features, seven studies included data on OS and four studies included data on DFS. The median follow-up of 7 studies is illustrated in Table 1. The patient population of most studies was rather heterogeneous with patients of all clinical stages. Twelve studies did not include patients with distant metastases. The percentage of patients with distant metastases in the residual studies was 50% to 87.9% [16, 30]. HPV status was detected in four studies. Subsites of primary tumors of twelve studies are illustrated in S1 Table including 413 oral cancers, 509 OPSCC and 177 at other sites. Information on treatment modalities was provided in only a single study. Therefore, treatment was not included in our statistical analysis.

**Table 1:** Main characteristics and results of the eligible studies.

Author	Year	Country	Number of Patients	Site	Tumor Stage (UICC)	Antibody	Cut-off score (H/L)	HPV status	Median follow-up
Chen YW [29]	2010	Taiwan	111	HNSCC	ND	ND, Abcam	ND	ND	75 months
Koukourakis MI [31]	2012	Greece	74	HNSCC	I-III	Rabbit mAb, EP1933Y, Abcam	5%	ND	24 months (4–80 months)
Michifuri Y [34]	2012	Japan	80	OSCC	I-III	Mouse mAb, clone 44, BD Pharmingen	2%	ND	ND
Xu J [17]	2012	USA	96	HNSCC	I-IV	N-19, Santa Cruz	0	ND	62 months (12–150 months)
Liu W [32]	2013	China	141	OSCC	ND	Rabbit mAb, ab52492, Abcam	5%	ND	66 months
Chen C [39]	2013	China	60	HNSCC	ND	ND, BD Biosciences	ND	ND	ND
Qian X [16]	2013	Germany	80	OPSCC	I-IV	Mouse mAb, clone 44; BD Biosciences	5%	Yes	ND
Ota N [35]	2014	Japan	90	OSCC	I-IV	Mouse mAb, clone 44; BD Biosciences	5%	ND	ND
Zhang M [36]	2014	USA	222	OPSCC	ND	Mouse mAb, clone 44; BD Biosciences	ND	Yes	ND
Huang CF [30]	2014	China	66	TSCC	I-IV	Polyclonal rabbit Ab, Proteintech Group Inc.	10%	ND	52 months (2–104 months)
Qian X [5]	2014	Germany	81	HNSCC	I-III	Mouse mAb, clone 44; BD Biosciences	5%	Yes	ND
Leinung M [37]	2015	Germany	48	HNSCC	ND	Rabbit mAb, ab52492; Abcam	ND	Yes	120 months
Martín M [33]	2016	Spain	57	LSCC	I-IV	ND, Abcam	ND	ND	42 months
de Moraes FP [38]	2016	Brazil	52	HNSCC	I-IV	EP1933Y, Abcam	10%	ND	ND

IHC: immunohistochemistry; HNSCC: head and neck squamous cell carcinoma; OSCC: oral squamous cell carcinomas; TSCC: tongue squamous cell carcinoma; LSCC: laryngeal squamous cell carcinoma; OSCC: oral squamous cell cancer; OPSCC: oropharyngeal squamous cell carcinomas; ND: not documented.

### Correlation of ALDH1 expression with clinicopathological parameters

**Fig 2:**
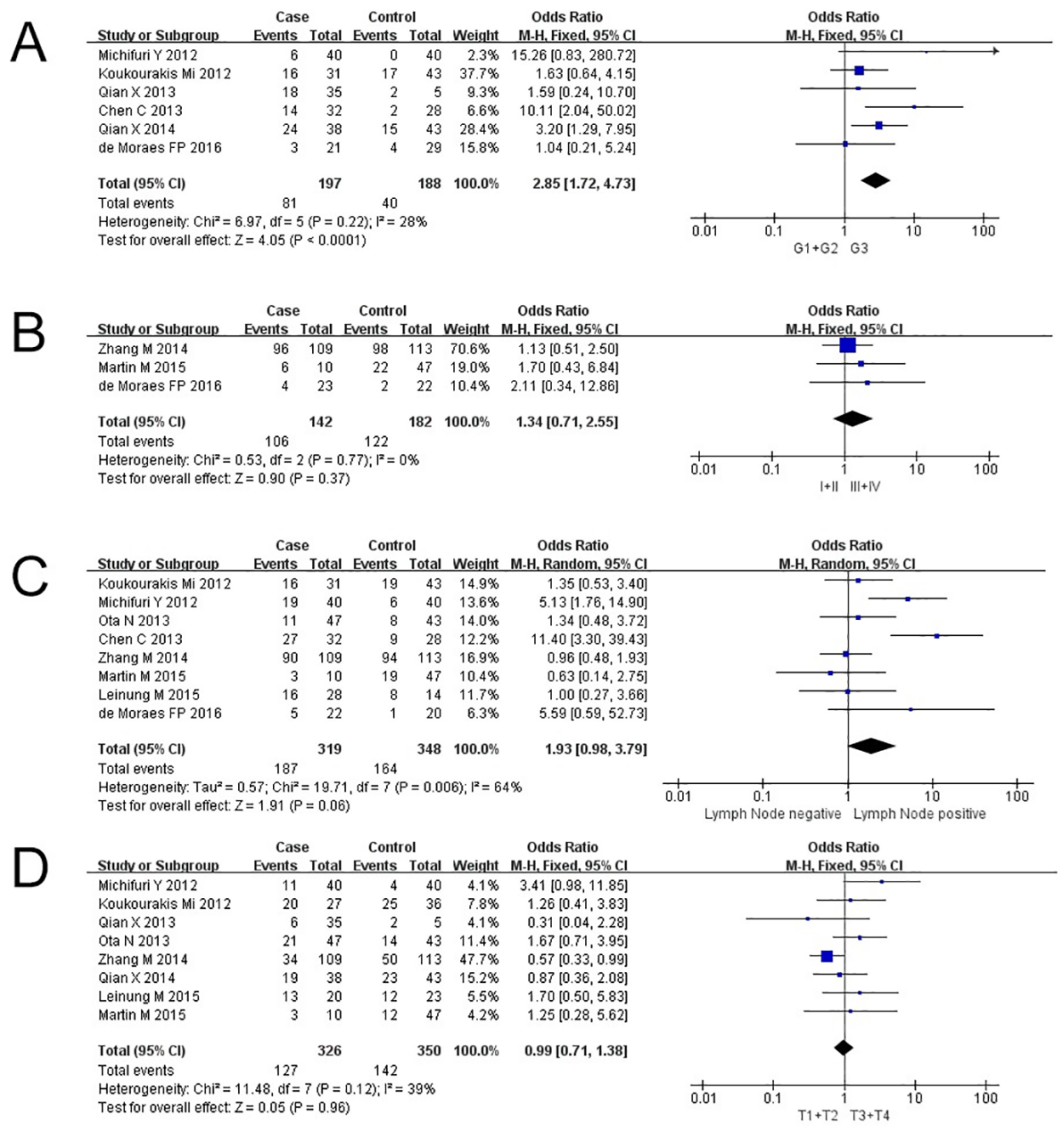
Forest plot describing the association between ALDH1 expression and odds ration (OR) for clinical pathological features. Forest plot of OR for the (A) differentiation grading, (B) clinical staging, (C) lymph node status, (D) tumor T stage of the ALDH1^+^ group compared to ALDH1^-^ group.

All studies used immunohistochemistry (IHC) with cut-off values between any staining to >10% positivity. Isoenzymes of ALDH1 are e.g., ALDH1A1, ALDH1A2, and ALDH1A3. The antibodies used, when described, were specific for all isoforms of ALDH1 in four studies, and ALDH1A1 in ten studies. Therefore, we used the term “ALDH1” in our analysis. The association of ALDH1 expression with clinicopathological parameters is illustrated in Fig 2. ALDH1 expression was associated with higher differentiation grade (G3 vs. G1+G2; OR = 2.85, 95% CI: 1.72–4.73, P<0.0001, fixed effect; Fig 2A) but not clinical stage (III+IV vs. I+II; OR = 1.34, 95% CI: 0.71–2.55, P = 0.37, fixed effect; Fig 2B). Sensitivity analysis showed stable results for differentiation (S1 Fig). Furthermore, expression of ALDH1 was not significantly associated with positive lymph node status (Pos vs. Neg; OR = 1.93, 95% CI: 0.98–3.79, P = 0.06, random effect; Fig 2C) or T-stage (T3+T4 vs. T1+T2; OR = 0.99, 95% CI: 0.71–1.38, P = 0.96, fixed effect; Fig 2D). ALDH1 expression was analyzed in four studies regarding the tumor sites. The studies of Koukourakis et al. and Ota et al. showed no significant relation of ALDH^low^ or ALDH^high^ expression to tumor sites.

### ALDH1 expression and disease-free survival

**Fig 3:**
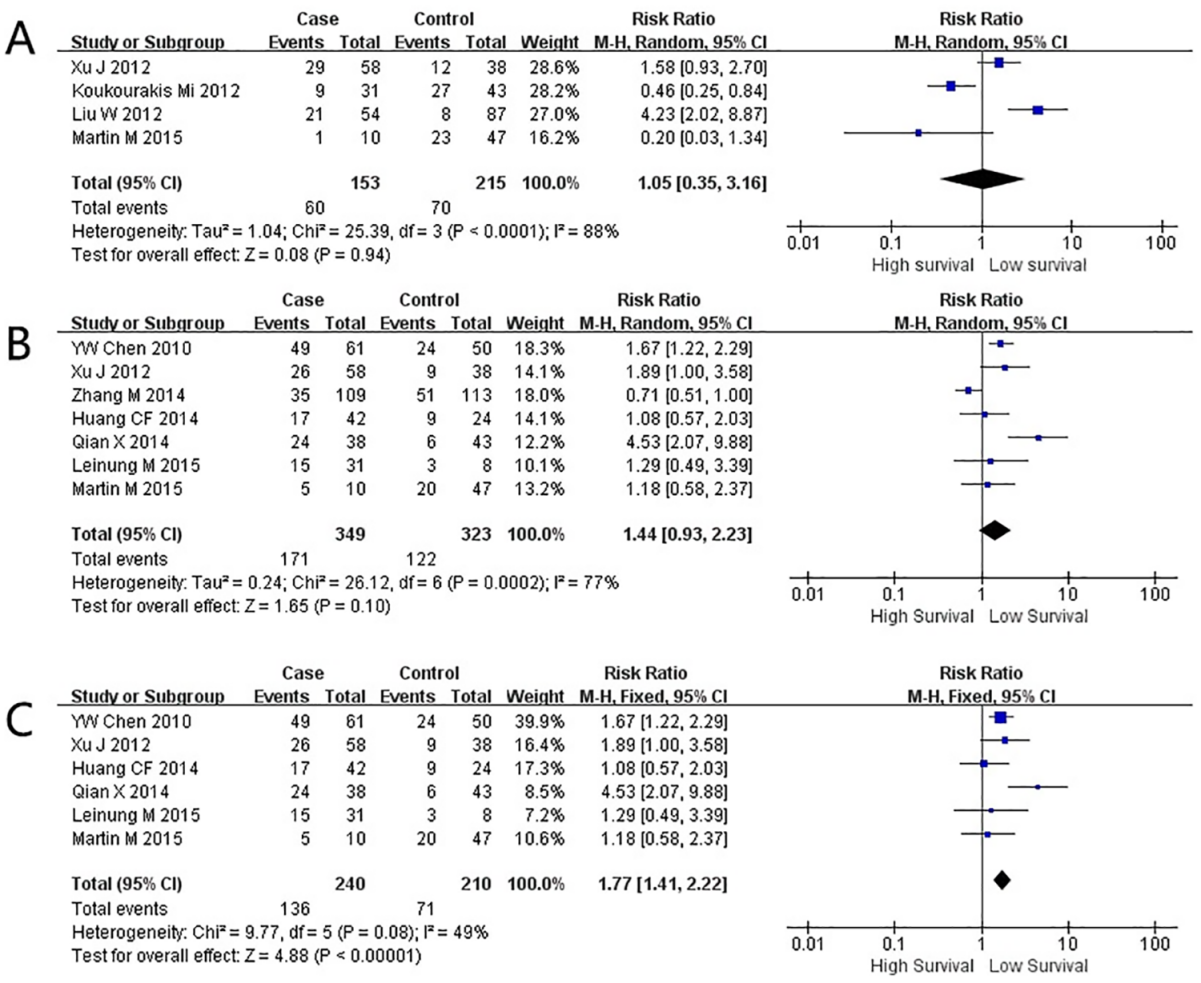
Meta-analysis for adjusted relative risk (RR) of the disease-free survival (DFS) or overall survival (OS) compared to the ALDH1^+^ subgroup. Forest plot of RR among included studies for the DFS of the ALDH1^+^ group compared to ALDH1^-^ group (A). Forest plot of RR for the OS (B) and adjusted OS (C) of the ALDH1^+^ group compared to ALDH1^-^ group.

DFS was investigated in 4 studies including 368 patients [17, 31–33]. We performed a meta-analysis on the DFS of ALDH^+^ and ALDH^-^ patients. There was no significant association between ALDH1 expression and DFS (RR = 1.05; 95% CI: 0.35–3.16; P = 0.94, random effect; Fig 3A), again with significant heterogeneity (P<0.0001; *I*^*2*^ = 88%). Then, we performed a sensitivity analysis where omission of each single article’s data did not result in obvious change in heterogeneity.

### ALDH1 expression and 5-year overall survival outcome

We analyzed the OS of 672 patients from seven articles [5, 17, 29, 30, 33, 36, 37]. Using the DerSimonian–Laird fixed effects model, the meta-analysis on the OS of ALDH^+^ and ALDH^-^ patients did not reach a statistical significance (RR = 1.44; 95% CI: 0.93–2.23, P = 0.10, random effect; Fig 3B). The forest plot showed a high level of heterogeneity, which was significant (I-squared and Q-test, P = 0.0002; *I*^*2*^ = 77%). In order to show the individual influence of each study on the over-all heterogeneity, we performed a sensitivity analysis by which each study was removed from the pool of studies once and *I*^*2*^ and *Q* value was calculated. We demonstrated that the study of Zhang *et al*. [36] was the source of heterogeneity (S2 Fig). The removal of the latter study from the pooled population resulted in a significantly reduced *I*^2^ value. Analysis of the modified patient population showed a conspicuous shorter OS in patients with positive ALDH1 expression (RR = 1.77, 95% CI: 1.41–2.22, P<0.0001, random effect), which was illustrated in the modified forest plot (Fig 3C).

### Risk of bias of included studies and publication bias

Regarding to the NOS scale, all included studies had a score greater than 5 indicating a high quality of each study (S2 Table). Begg’s funnel and Egger’s test were used to access the publication bias for each item (Table 2). Egger’s test showed no significant publication bias for T-stage (t = 0.70, P = 0.508), lymph node status (t = 1.01, P = 0.352), differentiation grade (t = 1.17, P = 0.306), OS (t = 0.83, P = 0.442) and DFS (t = -0.30, P = 0.792).

**Table 2:** Egger's test of funnel plot asymmetry.

Clinicopathological parameters	t value	df	p value
Tumor grade	1.17	5	0.306
Lymph node metastasis	1.01	7	0.352
Tumor T stage	0.70	7	0.508
Overall survival	0.83	6	0.442
Disease free survival	-0.30	3	0.792

df: degrees of freedom.

## Discussion

ALDH1-expression is a commonly used marker identifying putative CSC in a variety of epithelial tumors directly or indirectly, to identify cells displaying resistance to conventional cancer treatments [13]. In this meta-analysis, the presence of ALDH1-positive cells in HNSCC was analyzed regarding tumor grading, stage at first diagnosis, and clinical outcomes. We showed that the presence of ALDH1-positive cells within primary tumors was strongly associated with a higher grading, which means with more dedifferentiated tumors.

It is hypothesized that putative stem cells are not a defined subpopulation within the malignant cells. The development from CSC to more differentiated cancer cells at the same tumor site or after dissemination is believed to be continuous. Specific embryonic stem cell-associated transcriptional regulators and gene expression pathways appear to be more upregulated in poorly differentiated but not in well differentiated tumors [40]. On a cellular level, some of these transcriptional factors associated with embryonic stem cells, e. g., Sox2, Oct3/4, and Nanog were significantly upregulated in ALDH1-postive CSC [41]. Accordingly, low-grade tumors should contain mostly differentiated cells and few CSC, and high-grade tumors should contain highly dedifferentiated cells exhibiting stem cell-like features and a high percentage of CSC, a notion which we proved to be true in our meta-analysis. Recently, the dedifferentiation from non-CSC into ZsGreen-cODC-positive CSC, a subpopulation of ALDH1-positive cells, was found in both HPV-negative and HPV-positive HNSCC cell lines in response to radiation [42]. Moreover, HPV-positive HNSCC cells that survived radiation dedifferentiated in CSC at lower rates while HPV-negative HNSCC had a higher rate. This phenomenon not only demonstrates that dedifferentiation exists during radiotherapy and thus the capacity of CSC for repopulation, but also explains in part the better survival for patients with HPV-positive HNSCC. However, very little is known of whether a dedifferentiation from non-CSC to CSC on a cellular level is possible. What is known is that epithelial-mesenchymal transition (EMT) and its reversed process mesenchymal-epithelial transition is related to the acquisition of stem cell properties. It appears that EMT can reprogram differentiated mammary epithelial cells into less differentiated epithelial stem cells with mesenchymal traits [43]. It also has been demonstrated in HNSCC that overexpression of ALDH1 dramatically decreased expression of epithelial markers but increased expression of mesenchymal markers [4] leading to enhanced invasiveness and metastatic potential [44]. A better understanding of how and why CSC are induced and modulated local-regionally might suggest ways to target them.

HNSCC is used to describe all carcinomas arising from the stratified squamous epithelium lining the sinonasal tract, oral cavity, oropharynx, pharynx, and larynx. Histologically, squamous cell carcinoma is characterized by microscopic evidence of squamous differentiation and invasive growth. In most cases, the histological grading of these tumors is directly linked to the clinical stage at first diagnosis. At least for oral HNSCC, poorly differentiated tumors are more likely correlated with a positive nodal status, extracapsular spread, and perineural invasion [45]. Given the fact, that we found a clear association of ALDH1-expression with high histological grade, we expected an association of ALDH1 expression with clinical stage, T- and N-status. In a previous meta-analysis, Zhou *et al*. showed that the ALDH1 expression was significantly associated with lymph node metastasis, but not T-stage and the presence of distant metastases [46]. However, our analysis did not show significant impact of ALDH1 expression on disease stage. The discrepancy of the results may be due to the recent publications [33, 35, 36] enrolled in the dataset which have shown no significant correlations between lymph node metastasis and CSC. Although a significant correlation of ALDH1 CSC with nodal status was not concluded in this meta-analysis, in patterns of primary tumor and its corresponding nodal metastases, we (Qian *et al*.) showed that the proportion of ALDH1-expressing cells was significantly increased in nodal metastases compared to their corresponding primary tumors [16]. We also observed *in vitro* as a property of CSC an increased invasive capacity and expression of EMT-markers and decreased expression of adhesion molecule E-cadherin [4]. In addition, the EMT process can be reversed by transfer of miR34a-mimics into HNSCC cells [41]. We are currently planning a morphoproteomics-based prospective study to further investigate the outcome of HNSCC regarding CSC and EMT markers in order to tailor the possibility of biomarker-directed therapy.

In addition, HPV status may also be responsible for discrepant results. There was no correlation between ALDH1 expression and nodal metastases when only HPV^-^ tumors were included in the study of Leinung *et al*. [37]. The study by Zhang *et al*. [36] showed that HPV16^+^ OPSCC had a higher intrinsic CSC pool than HPV^-^ OPSCC and no correlation was found between nodal status and ALDH1 positive CSC for the whole cohort. Because the authors did not perform the subgroup analysis based on HPV status, the frequency of CSC in HPV^+^ and HPV^-^ tumors was not documented. According to the recent report of global incidence of HPV infection in head and neck cancer [47], around 30% of OPSCC are caused by HPV, followed by oral cavity and larynx. The majority of subsites included in this meta-analysis are OPSCC, oral cavity and larynx. Thus, discrimination on HPV status to assess the effect of ALDH1 expression on lymph node metastasis is helpful to confirm or rebut this hypothesis.

The weakness of our meta-analysis is mainly the heterogeneity of studies and lack of clinical data. We could not find a statistical difference in DFS, which is a discrepancy compared to a previously published evaluation [46]. Indeed, several factors may account for the discrepancy of survival between two datasets. While the study by Liu *et al*. showed a clear signal for ALDH1 being associated with a shorter PFS, Martin *et al*. found the opposite result. Here it is important to note that the study by Liu *et al*. only included carcinomas of oral cavity [32]. Martin *et al*., on the other hand, included only laryngeal carcinomas with a portion of 26% stage IV patients [33]. Anatomic site, stage and histological characteristics are considered to be of importance for risk stratification of HNSCC [48–50]. As shown in a recent report by The Cancer Genome Atlas Network [51], increased expression of oxidative stress response genes associated with chemo-resistance was found in laryngeal carcinomas while oral cavity tumors exhibit lower expression of genes related to DNA repair. Other confounding factors may include different ALDH1 testing methods used in each study and different treatment protocols. The prognostic impact of ALDH1 positivity on OS in HNSCC patients has been detected in seven of fourteen studies. When one source study was excluded, there was a significant impact of ALDH1 positivity on OS. Again, the observed heterogeneity of the patient populations is more likely to be responsible for the equivocal findings. The study by Zhang et al. [36] included exclusively HPV^+^ LA tumors which was associated with a favorable outcome than HPV^-^ LA tumors [52]. Also, differences do exist in the CSC pool of HPV^+^ tumors and HPV^-^ tumors [16, 42]. There are disagreements between the study of Zhang *et al*. where HPV^+^ HNSCC cells had a greater intrinsic CSC pool and other studies which showed a significantly lower frequency of CSCs in HPV^+^ tumors [42, 53]. In addition, because the patient samples enrolled in the Zhang study were microarrays, there would be limitations to conclude the results based on the selected tumor cores. They observed ALDH1 staining in >50% of the tumor cells in selected HPV^-^ and HPV16^+^ OPSCC tumor cores. However, in our own study, the ALDH1 staining ranged from 0 to >50% in primary tumor and its corresponding nodal metastases. We also found higher ALDH1 expression grades and negative HPV status for primary tumors but not for metastases. These findings are consistent with studies in HNSCC cell lines which have shown HPV^+^ cell lines had lower numbers of CSCs [41] and inversely correlated with radiosensitivity [42]. Although these studies seem contradictory, it is worth noting that a small proportion of less radioresistant CSCs being identified in HPV-induced HNSCC were positively associated with decreased local regional control [54] indicating a contribution of CSC to the decreased local regional control in HPV^+^ tumors. The reason behind the observed differences warrants further investigations.

It is clear that CSC play crucial roles in tumorigenesis based on earlier observations, but currently its clinical relevance such as its proportions in pre- and post-treatment stage, response to treatment modalities and outcomes is still limited. The data revealed that ALDH1 expression is associated with tumor histology independent of HPV status indicating ALDH1-positive CSC as a potentially valuable tool in clinical management of HNSCC. The data also revealed the potentially prognostic value of ALDH1-positive CSC in HNSCC. It is necessary to further investigate the value of CSC presence as markers in stratified HNSCC subgroups based on factors such as HPV-association or tumor site. Treatment modalities targeting CSC populations in order to improve therapy are also currently of interest. Kulsum *et al*. demonstrated that ALDH1A1 inhibition improved the effectiveness of cisplatin treatment, reduced the migration rate, self-renewal capacity and tumorigenicity of cancer cells *in vitro*. Further, an *ex vivo* study continuously showed that ALDH1A1-specific inhibitor in combination with cisplatin significantly decreased the proliferation of cells as compared to individual treatment [18]. Thus, strategies to introduce ALDH1A1-specific inhibitors to current chemoradiotherapy regimens, in an effort to target CSC and treatment-induced reprogramming, may become an ideal treatment to achieve better curative effects.

In conclusion, we have presented that ALDH1 expression is associated with tumor histology and has potential prognostic value independent of etiologies such as chronic alcohol, tobacco abuse, and HPV status in the meta-analysis. Additional limitations of our meta-analysis are the number of included articles and that the sample size of each study was rather small. There was heterogeneity of data among outcomes. We acknowledge that because of the limited data for our question of interest such as HPV status and tumor subsites were not discriminated, the strength of our conclusion is limited. Given the massive biases built in to case-control studies, ALDH1 as a putative biomarker should (at most) be presented as speculative. Future large-scale prospective studies to establish and validate the prognostic value of ALDH1 are necessary.

## Supporting information

S1 Fig Sensitivity analysis of ALDH1 expression with differentiation of HNSCC tissues. (TIF)

S2 Fig Sensitivity analysis of ALDH1 expression with overall survival of HNSCC. (TIF)

S1 Table Distribution of primary tumor sites of the eligible studies. (DOC)

S2 Table Newcastle-Ottawa quality assessment scale of included studies. (DOCX)

S1 File Search terms and the number of studies identified from Pubmed. (DOCX)

S2 File PRISMA checklist. (DOC)
