# Supplementary figures and images for: Aldehyde dehydrogenase 1 isoenzyme expression as a marker of cancer stem cells correlates to histopathological features in head and neck cancer: A meta-analysis
Source: PLoS One. 2017 Nov 7;12(11):e0187615. doi: 10.1371/journal.pone.0187615 (PMC5675382; doi:10.1371/journal.pone.0187615)

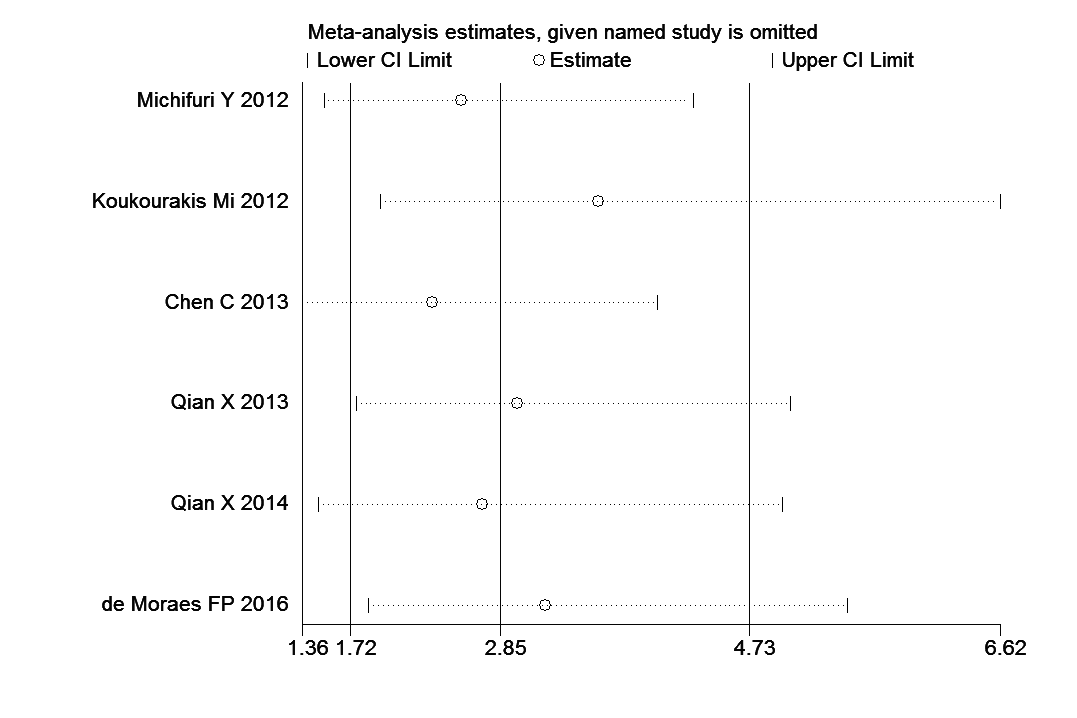

Supplement: S1 Fig — (TIF) [file pone.0187615.s001.tif]

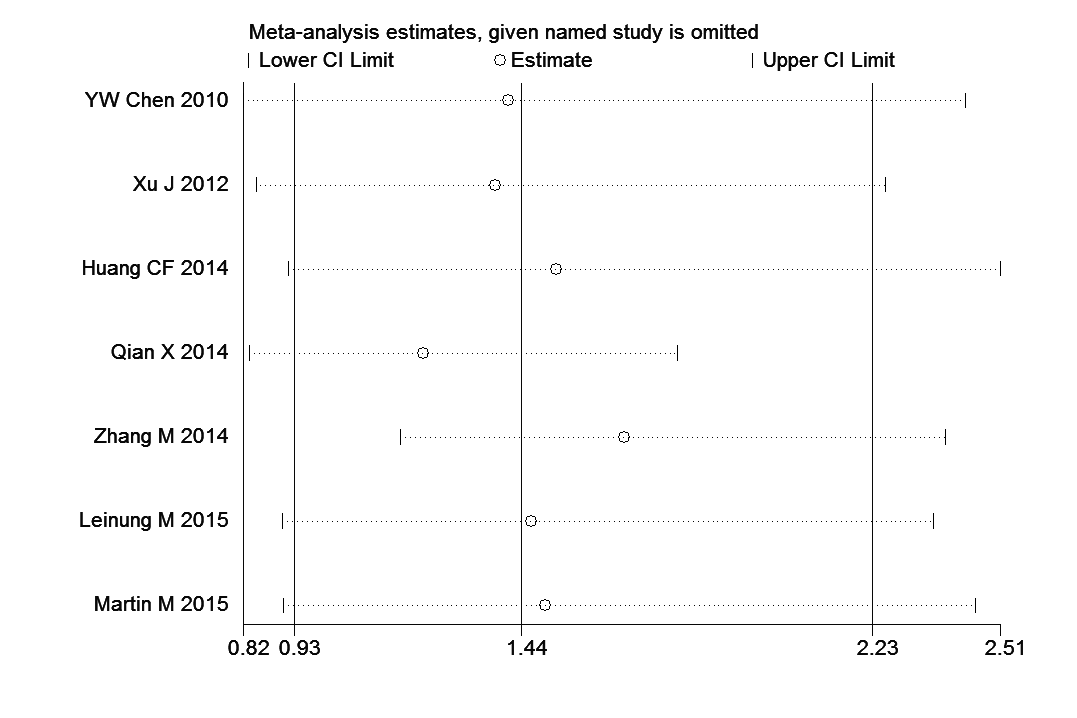

Supplement: S2 Fig — (TIF) [file pone.0187615.s002.tif]
