# Supplementary material for: Aldehyde dehydrogenase 1 isoenzyme expression as a marker of cancer stem cells correlates to histopathological features in head and neck cancer: A meta-analysis
Source: PLoS One. 2017 Nov 7;12(11):e0187615. doi: 10.1371/journal.pone.0187615 (PMC5675382; doi:10.1371/journal.pone.0187615)
Supplement: S1 Table — (DOC) [file pone.0187615.s003.doc]

**S1 Table.** **Distribution of primary tumor sites of the eligible studies.**

| **Author** | **Patient number** | **Tumor site** | | | | | | |
| --- | --- | --- | --- | --- | --- | --- | --- | --- |
|  |  | Oral cavity | Oropharynx | Hypopharyx | Larynx | Nasopharynx | Nodal | Parotid |
| Chen YW [29] | 111 | ND | ND | ND | ND | ND | ND | ND |
| Koukourakis MI [31] | 74 | - | 13 | 6 | 37 | 8 | 8 | 2 |
| Michifuri Y [34] | 80 | 80 | - | - | - | - | - | - |
| Xu J [17] | 96 | 42 | 29 | - | 25 | - | - | - |
| Liu W [32] | 141 | 141 | - | - | - | - | - | - |
| Chen C [39] | 60 | 22 | 18 | 20 | - | - | - | - |
| Qian X [16] | 80 | - | 80 | - | - | - | - | - |
| Ota N [35] | 90 | 90 | - | - | - | - | - | - |
| Zhang M [36] | 222 | - | 222 | - | - | - | - | - |
| Huang CF [30] | 66 | - | 66 | - | - | - | - | - |
| Qian X [5] | 81 | 2 | 65 | 1 | 13 | - | - | - |
| Leinung M [37] | 48 | ND | ND | ND | ND | ND | ND | ND |
| Martín M [33] | 57 | - | - | - | 57 | - | - | - |
| de Moraes FP [38] | 52 | 36 | 16 | - | - | - | - | - |

ND: not documented.
