# Supplementary material for: Aldehyde dehydrogenase 1 isoenzyme expression as a marker of cancer stem cells correlates to histopathological features in head and neck cancer: A meta-analysis
Source: PLoS One. 2017 Nov 7;12(11):e0187615. doi: 10.1371/journal.pone.0187615 (PMC5675382; doi:10.1371/journal.pone.0187615)
Supplement: S2 Table — (DOCX) [file pone.0187615.s004.docx]

**S2 Table.** **Newcastle-Ottawa quality assessment scale of included studies.**

| **Reference** | **Selection** | | | | **Comparability** | **Exposure** | | | **Score** |
| --- | --- | --- | --- | --- | --- | --- | --- | --- | --- |
| Authors, year | (1) | (2) | (3) | (4) | (5) | (6) | (7) | (8) |  |
| Chen Y, 2010 | **☆** | **☆** | **☆** | **☆** | **☆** | - | **☆** | - | 6 |
| Koukourakis M, 2012 | **☆** | **☆** | **☆** | **☆** | **☆** | **☆** | **☆** | - | 7 |
| Michifuri Y, 2012 | **☆** | - | **☆** | **☆** | **☆** | - | **☆** | - | 5 |
| Xu J, 2012 | **☆** | **☆** | **☆** | **☆** | **☆☆** | - | **☆** | - | 7 |
| Liu W, 2012 | **☆** | - | **☆** | **☆** | **☆☆** | **☆** | **☆** | - | 7 |
| Chen C, 2013 | **☆** | - | **☆** | **☆** | **☆** | - | **☆** | - | 5 |
| Qian X, 2013 | **☆** | - | **☆** | **☆** | **☆☆** | **☆** | **☆** | - | 7 |
| Ota N, 2013 | **☆** | - | **☆** | **☆** | - | **☆** | **☆** | - | 5 |
| Zhang M, 2014 | **☆** | - | **☆** | **☆** | **☆** | - | **☆** | - | 5 |
| Huang C, 2014 | **☆** | - | **☆** | **☆** | **☆☆** | **☆** | **☆** | - | 7 |
| Qian X, 2014 | **☆** | **☆** | **☆** | **☆** | **☆☆** | **☆** | **☆** | - | 8 |
| Martín M, 2015 | **☆** | - | **☆** | **☆** | **☆☆** | - | **☆** | **☆** | 7 |
| Leinung M, 2015 | **☆** | **☆** | **☆** | **☆** | **☆** | - | **☆** | - | 6 |
| de Moraes FP, 2016 | **☆** | **☆** | **☆** | **☆** | **☆** | - | **☆** | - | 6 |

Notes: (1) Is the case definition adequate; (2) Representativeness of the cases; (3) Selection of controls; (4) Definition of controls; (5) Comparability of cases and controls on the basis of the design or analysis; (6) Ascertainment of exposure; (7) Same method of ascertainment for cases and controls; (8) Non-response rate.
