# Supplementary material for: Aldehyde dehydrogenase 1 isoenzyme expression as a marker of cancer stem cells correlates to histopathological features in head and neck cancer: A meta-analysis
Source: PLoS One. 2017 Nov 7;12(11):e0187615. doi: 10.1371/journal.pone.0187615 (PMC5675382; doi:10.1371/journal.pone.0187615)
Supplement: S1 File — (DOCX) [file pone.0187615.s005.docx]

**Search strategy**

(A) Pubmed

70 articles

ALDH1

aldehyde dehydrogenase 1 [MeSH Terms] OR aldehyde dehydrogenase 1 [Title/Abstract] OR ALDH1 [Title/Abstract]

HNSCC

(head and neck squamous cell[Title/Abstract] OR oral[Title/Abstract] OR laryngeal[Title/Abstract] OR pharyngeal[Title/Abstract] OR tongue[Title/Abstract] OR oropharyngeal[Title/Abstract]) AND (carcinoma [MeSH Terms] OR carcinoma [Title/Abstract] OR neoplasm[Title/Abstract] OR cancer[Title/Abstract])

Total search

(aldehyde dehydrogenase 1 [MeSH Terms] OR aldehyde dehydrogenase 1 [Title/Abstract] OR ALDH1 [Title/Abstract]) AND (head and neck squamous cell[Title/Abstract] OR oral[Title/Abstract] OR laryngeal[Title/Abstract] OR pharyngeal[Title/Abstract] OR tongue[Title/Abstract] OR oropharyngeal[Title/Abstract]) AND (carcinoma [MeSH Terms] OR carcinoma [Title/Abstract] OR neoplasm[Title/Abstract] OR cancer[Title/Abstract])

(B) Embase

118 articles

ALDH1

'aldehyde dehydrogenase 1'/exp OR ‘aldehyde dehydrogenase 1’:ti,ab OR ‘ALDH1’:ti,ab

HNSCC

(‘head and neck squamous cell’:ti,ab OR ‘oral’:ti,ab OR ‘laryngeal’:ti,ab OR ‘pharyngeal’:ti,ab OR ‘tongue’:ti,ab OR ‘oropharyngeal’:ti,ab) AND (‘carcinoma”/exp OR ‘carcinoma’;ab,ti OR ‘cancer’;ab,ti OR ‘neoplasm’;ab,ti)

Total search

('aldehyde dehydrogenase 1'/exp OR ‘aldehyde dehydrogenase 1’:ti,ab OR ‘ALDH1’:ti,ab) AND (‘head and neck squamous cell’:ti,ab OR ‘oral’:ti,ab OR ‘laryngeal’:ti,ab OR ‘pharyngeal’:ti,ab OR ‘tongue’:ti,ab OR ‘oropharyngeal’:ti,ab) AND (‘carcinoma”/exp OR ‘carcinoma’;ab,ti OR ‘cancer’;ab,ti OR ‘neoplasm’;ab,ti)

(C) Cochrane Library

3 articles

ALDH1

[mh " aldehyde dehydrogenase 1"] OR “aldehyde dehydrogenase 1”; ti,ab,kw OR “ALDH1”;ti,ab,kw

HNSCC

(‘head and neck squamous cell’:ti,ab,kw OR ‘oral’:ti,ab,kw OR ‘laryngeal’:ti,ab,kw OR ‘pharyngeal’:ti,ab,kw OR ‘tongue’:ti,ab,kw OR ‘oropharyngeal’:ti,ab,kw) AND ([mh " carcinoma"] OR ‘carcinoma’;ab,ti,kw OR ‘cancer’;ab,ti,kw OR ‘neoplasm’;ab,ti,kw)

Total search

([mh " aldehyde dehydrogenase 1"] OR “aldehyde dehydrogenase 1”; ti,ab,kw OR “ALDH1”;ti,ab,kw) AND (‘head and neck squamous cell’:ti,ab,kw OR ‘oral’:ti,ab,kw OR ‘laryngeal’:ti,ab,kw OR ‘pharyngeal’:ti,ab,kw OR ‘tongue’:ti,ab,kw OR ‘oropharyngeal’:ti,ab,kw) AND ([mh " carcinoma"] OR ‘carcinoma’;ab,ti,kw OR ‘cancer’;ab,ti,kw OR ‘neoplasm’;ab,ti,kw)
