# Supplementary material for: Aldehyde dehydrogenase 1 isoenzyme expression as a marker of cancer stem cells correlates to histopathological features in head and neck cancer: A meta-analysis
Source: PLoS One. 2017 Nov 7;12(11):e0187615. doi: 10.1371/journal.pone.0187615 (PMC5675382; doi:10.1371/journal.pone.0187615)
Supplement: S2 File — (DOC) [file pone.0187615.s006.DOC]

| **Section/topic** | **#** | **Checklist item** | **Reported on page #** |
| --- | --- | --- | --- |
| **TITLE** | | |  |
| Title | 1 | A Meta-Analysis | 1 |
| **ABSTRACT** | | |  |
| Structured summary | 2 | There is a lack of predictive biomarkers that can identify patients with head and neck squamous cell carcinoma (HNSCC) who will experience treatment failure and develop drug resistance, recurrence, and metastases. Cancer stem-like cells (CSC) were identified as a subset of cells within the tumor in a variety of solid tumors including HNSCC. CSC are considered the tumor-initiating population responsible for recurrence or metastasis and are associated with therapy resistance. This meta-analysis including fourteen studies with altogether 1258 patients updates and summarizes all relevant data on the impact of ALDH1^+^ CSC on the prognosis of HNSCC and its association with clinicopathological parameters. ALDH1 expression is highly correlated with tumor differentiation (G3 vs. G1+G2; odds ratio=2.85. 95% CI: 1.72-4.73, P<0.0001) and decreased overall survival (relative risk=1.77. 95% CI: 1.41-2.22, P<0.0001) if one out of seven studies was excluded because of heterogeneity. These findings provide insights into the understanding of more aggressive tumor phenotypes and also suggest that the prognostic value provided by HNSCC-subtyping by CSC frequency warrant further clinical investigation. | 2 |
| **INTRODUCTION** | | |  |
| Rationale | 3 | CSC initiate metastasis formation and recurrence after initial therapy. It is therefore being important to characterize and evaluate the ALDH1A1 CSC compartment in HNSCC. | 3- 5 |
| Objectives | 4 | In order to assess clinical and biological characteristics such as histological appearance, lymph node metastasis and patient’s survival in relation to ALDH1 expression in HNSCC. | 3- 5 |
| **METHODS** | | |  |
| Protocol and registration | 5 |  |  |
| Eligibility criteria | 6 | 1. Patients with HNSCC; 2. Correlations between ALDH1 overexpression and histopathological parameters, overall survival (OS) or disease-free survival (DFS) of HNSCC patients were analyzed. | 5-6 |
| Information sources | 7 | PubMed (1966–2016), EMBASE (1980–2016), the Cochrane Library (1996–2016) and the WanFang databases (1988–2016) were searched. | 5 |
| Search | 8 | The following combined search term was used: (ALDH1” or “aldehyde dehydrogenase 1”) AND (“head and neck squamous cell carcinoma” or “oral cancer” or “laryngeal cancer” or “pharyngeal cancer” or “tongue cancer” or “oropharyngeal cancer). We combined the term appropriately with MeSH Terms and used an appropriate adjustment for different databases. | 5 |
| Study selection | 9 | A total of 155 studies were retrieved. After reading the titles and abstracts, 110 studies were excluded. 45 full text studies were carefully reviewed (excluded for reviews and meta-analyses, no control group and completely irrelevant**).** Finally, 14 studies were included for our analysis. | 7- 8 |
| Data collection process | 10 | Pivotal data were extracted from all eligible publications independently by two of the authors. The difference of opinions was discussed and resolved with the corresponding authors. | 6-7 |
| Data items | 11 | Patients’ characteristics such as average age, gender, TNM stage and survival data were extracted. | 6-8 |
| Risk of bias in individual studies | 12 | The Newcastle-Ottawa Scale (NOS), a risk of bias assessment tool for observational studies, was used to evaluate the methodological quality of included studies. The quality assessment values ranged from 0 to 9 points. There are three categories including selection (4 points), comparability (2 points), and exposure (3 points). The result was an overall risk of bias rating of each study and score ≥5 is considered high quality. Two independent reviewers evaluated the risk of bias for each study. | 6 |
| Summary measures | 13 | Results were expressed as odds ratios (OR), risk ratio (RR) and 95% confidence intervals (95% CI). | 7 |
| Synthesis of results | 14 | A fixed-effects model was adopted in the case of no evidence of significant heterogeneity (P > 0.05 and I^2^ < 50%); otherwise, a random-effects model was used. If heterogeneity was explored and sensitivity analyses were performed. We conducted a sensitivity analysis to identify the study with the most heterogeneity. | 7 |

Page 1 of 2

| **Section/topic** | **#** | **Checklist item** | **Reported on page #** |
| --- | --- | --- | --- |
| Risk of bias across studies | 15 | Egger’s test was performed to evaluate the publication bias (P < 0.05 was considered significant). | 7 |
| Additional analyses | 16 | Sensitive analysis was also performed to evaluate the influences of individual studies on the final effect size. When some studies were omitted, if no decreases in heterogeneity were observed, a qualitative systematic review method was used to describe the results. All P values were 2-sided, and P < 0.05 was considered significant. | 7 |
| **RESULTS** | | |  |
| Study selection | 17 | A total of 155 studies were retrieved. After reading the titles and abstracts, 110 studies were excluded. 45 full text studies were carefully reviewed and the included studies were requested to meet the following criteria: (1) patients with HNSCC; (2) correlations between ALDH1 overexpression and histopathological parameters, OS or DFS of HNSCC patients were analyzed. Finally, 14 studies were included for our analysis. | 7 |
| Study characteristics | 18 | Characteristics of each study were illustrated in Table 1. | 9 |
| Risk of bias within studies | 19 | The NOS was performed to assess the bias of the included articles. | 11 |
| Results of individual studies | 20 |  |  |
| Synthesis of results | 21 | The results of the meta-analysis were showed in Figure 2 and Figure 3. ALDH1 expression was associated with higher differentiation grade (G3 vs. G1+G2; OR=2.85, 95% CI: 1.72-4.73, P<0.0001, fixed effect; Fig. 2A) but not clinical stage (III+IV vs. I+II; OR=1.34, 95% CI: 0.71-2.55, P=0.37, fixed effect; Fig. 2B; Sensitivity analysis showed the result for differentiation was stable), positive lymph node status (Pos vs. Neg; OR=1.93, 95% CI: 0.98-3.79, P=0.06, random effect; Fig. 2C) and T-stage (T3+T4 vs. T1+T2; OR=0.99, 95% CI: 0.71-1.38, P=0.96, fixed effect; Fig. 2D). For survival analysis, ALDH1 expression was no significant association between ALDH1 expression and DFS (RR=1.05; 95% CI: 0.35-3.16; P=0.94, random effect; Fig. 3A) but significant related with decreased overall survival (RR=1.77. 95% CI: 1.41-2.22, P<0.0001) if one of seven studies was excluded because of heterogeneity. | 8- 11 |
| Risk of bias across studies | 22 | Egger’s test (as shown in Table 2) was used to evaluate the publication bias of included studies. Publication bias was not found in our study. | 11- 12 |
| Additional analysis | 23 | Sensitivity analyses, in which one study was removed at a time, were performed to evaluate the stability of the results. | 8- 11 |
| **DISCUSSION** | | |  |
| Summary of evidence | 24 |  |  |
| Limitations | 25 | Inevitably, our meta-analysis has some limitations. The number of the included articles and the sample size was small. There was heterogeneity of data among outcomes. | 17- 18 |
| Conclusions | 26 | In conclusion, our data demonstrate that ALDH1 expression is associated with tumor histology and has potential prognostic value for HNSCC independent of etiologies of HNSCC such as chronic alcohol, tobacco abuse, and HPV status. | 17- 18 |
| **FUNDING** | | |  |
| Funding | 27 | The study was supported by a grant from the Opening Project of Zhejiang Provincial Top Key Discipline of Clinical Medicine (grant No. LKFJ008) to XQ. | 18 |

*From:*  Moher D, Liberati A, Tetzlaff J, Altman DG, The PRISMA Group (2009). Preferred Reporting Items for Systematic Reviews and Meta-Analyses: The PRISMA Statement. PLoS Med 6(7): e1000097. doi:10.1371/journal.pmed1000097

For more information, visit: **www.prisma-statement.org**.

Page 2 of 2
